# New Disinfectant for Dressing of Gangrenous Wounds, Ulcers, Etc.

**Published:** 1860-05

**Authors:** 


*On a new Disinfectant for the Dressing of Gangrenous Wounds, Ulcers, &c.*—The medical faculty of Paris had their interest greatly excited, lately, by the recommendation, by Dr. Demaux and M. Corne (a veterinary surgeon), of a disinfectant which seems to answer its purpose admirably. These gentlemen brought their discovery before the "Academie des Sciences," who appointed Messrs. Cheveul, Velpeau and Cloquet (men whose names are familiar to most of our readers,) a committee to report upon it. On the 18th of July, 1849, this committee handed in the following report, which we have translated, for the benefit of our readers, from the "Gazette Medicale de Paris.":

"We have the honor to submit to the Academy of Sciences the results of numerous and varied experiments, made in common; first, in the private practice of one of us, and afterwards equally repeated in common, in the 'Charite Hospital,' in the wards of Professor Velpeau. We confine ourselves to state, in the following propositions, the facts which have, in the main, been confirmed by him, by his students, and by the physicians who attend regularly his lectures:

"1. A gangrenous wound, with abundant and fœtid suppuration, submitted to this new mode of dressing, was at once rid of all disagreeable odor,

"2. After 24 or even 36 hours, the dressing linen of the wound of bad character did not smell more offensively than that of a simple fracture.

"3. An ulcerated cancer, with an ichorous discharge, and with that fœtid odor peculiar to all cancers, was submitted to this dressing. The bad odor disappeared almost instantaneously, and did not return, so long as the dressing was used.

"4. Old ulcers of the legs, dressed in that way, were, with the same facility, deprived of all bad odor.

"5. Dressing linen soaked with fœtid pus, or poultices impregnated with such pus, lost immediately their bad odor when brought into contract with the disinfectant mixture.

"6. Infected liquids—products of gangrene—clots of decomposed blood—sphacelated tissue, in a very advanced state of putrefaction, were at once disinfected by this new remedy.

"The action of the disinfecting substance seems to arrest decomposition; it keeps away the insects and prevents certainly, the production of worms. It may be employed for many other purposes, which we will not mention here. And all these results are obtained by means at once simple, and of easy application; and with substances which can be found every where at a low price. The price of the disinfecting matter, all prepared, is in Paris, about a franc (18 cents,) for 50 kilogrammes (125℔ troy). It is in the form of powder, of a grayish color, more or less dark, according to the purity of the ingredients and their proportions, exhaling a slightly bituminous odor. Its composition is as follows:

"Of the common plaster of Paris (reduced to the finest powder), 100 parts; of coal tar (produced by the distillation of coal for the manufacture of gas), 1 to 3 parts,

"These substances can easily be mixed in a mortar.

"The application of this substance for the dressing of wounds, requires some peculiar preparation which we will show presently. If a certain quantity of the above powder be mixed with olive oil, a paste pomatum or ointment may be obtained (according to the quantity of oil used), that will keep any length of time if put up well. This salve is of a dark brown color and bituminous odor.

"The oil binds the powder, without dissolving it, in such a way as to preserve its property (by the gradual elimination of the oil,) of absorbing pus, if brought into contact with suppurating wounds.

"The consistency which it acquires when employed by itself, or with oil as an ointment, is never such as to give the patient any discomfort, or do the wound any injury. The application may be made approximately or immediately, according to the end desired. The immediate application does not produce the least pain. It has even a detersive power, and favors cicatrization.

"This mode of dressing, has the double property of disinfecting pus, and other morbid products, and of absorbing them. This last circumstance is so much more important, as it enables us to dispense with the use of lint (charpie)."—*Oglethorpe Medical & Surgical Journal.*

